# Effects of Exercise Compression Stockings on Anterior Muscle Compartment Pressure and Oxygenation During Running: A Randomized Crossover Trial Conducted in Healthy Recreational Runners

**DOI:** 10.1007/s40279-019-01103-y

**Published:** 2019-05-15

**Authors:** Kajsa Rennerfelt, Sophia Lindorsson, Helena Brisby, Adad Baranto, Qiuxia Zhang

**Affiliations:** Department of Orthopedics, Institute of Clinical Sciences, Sahlgrenska University Hospital, Sahlgrenska Academy, University of Gothenburg, Bruna Stråket 11B, Floor 4, 413 45 Gothenburg, Sweden

## Abstract

**Background:**

Exercise compression garments have increased in popularity among athletes at all levels during the last 10 years. However, the scientific grounds for this are unclear. The purpose of the present study was to examine the effect of wearing exercise compression stockings (CS) on the anterior compartment pressure, oxygenation of the tibialis anterior muscle, and early blood biomarkers change for muscle damage during a 10-km treadmill run in healthy subjects.

**Methods:**

Twenty healthy subjects completed two identical treadmill runs, with or without CS. The subjects were randomized regarding the order in which the sessions were performed. Intramuscular pressure (IMP) and muscle oxygenation in the one leg were continuously measured before, during, and after running sessions. Blood samples were collected just before and directly after these sessions and analyzed for myoglobin and creatine kinase concentrations.

**Results:**

The use of CS during running resulted in significantly higher IMP (by 22 ± 3.1 mmHg on average) and lower tissue oxygenation index (by 11 ± 1.8%) compared to running without CS (*p* < 0.001). In addition, the Δ change in median serum myoglobin concentration measured before and after running was significantly higher when CS were used: 58 (9‒210) µg/L as compared to 38 (0‒196) µg/L with no CS (*p* = 0.04). No difference in post-running early serum creatine kinase concentration was observed between using CS and not using CS.

**Conclusion:**

Wearing exercise CS during and following a 10-km treadmill run elevated IMP and reduced muscle tissue oxygenation in the anterior compartment of healthy runners. Furthermore, the use of exercise CS did not prevent early exercise-induced muscle damage, as measured by serum biomarkers.

## Key Points

**Table:** 

In the individuals tested, the use of exercise compression stockings during a 10-km treadmill run caused on average a 22-mmHg increase in intramuscular pressure compared to running without compression stockings, and the increase in intramuscular pressure subsequently led to a measurable reduction in muscle tissue oxygenation.
Wearing exercise compression stockings did not prevent exercise-induced muscle damage, as serum myoglobin significantly increased compared to running without compression stockings.
Healthy runners do not gain any circulatory benefits from wearing exercise compression stockings during a 10-km treadmill run..

## Introduction

During the past years, certain types of exercise compression stockings (CS) have been launched as highly technological sportswear with the purpose of increasing performance during running, both in elite and recreational runners, with a positive effect on muscle function [1]. CS for the lower extremities were originally developed for patients suffering from chronic venous insufficiency in order to increase the return of blood to the heart and reduce peripheral pooling and edema [2]. The technological CS on the sports market are claimed to optimize the local blood circulation and to decrease muscular waste products during activity, and also to improve recovery following exercise [3]. The interest in fitness and health activities is constantly growing. According to an analysis by the US national trade organization for the sport, Running USA, running participation in road races was almost 18.3 million registrants in 2017 [4]. Many of these recreational runners are interested in using CS, as they are marketed with the claim that they can improve runners’ performance.

There are conflicting data on how CS affect muscle function during exercise, in terms of oxygenation and muscle breakdown, and whether or not CS may be beneficial to healthy individuals. The studies with positive results regarding the benefits of wearing CS during exercise have found that CS increase tissue oxygenation and reduce venous pooling [5–7]. Kraemer et al. [8] showed that a compression garment reduced the level of muscle damage markers, such as serum creatine kinase (CK), after exercise. It has also been found that the use of CS following exercise may reduce perceived muscle soreness, reduce swelling, and promote recovery [9]. However, other studies have found negative results regarding the effects of wearing CS during exercise on oxygen uptake, heart rate, blood pressure, cardiac output, blood velocity in the popliteal artery, calf muscle tissue oxygenation, arterial lactate concentration, oxygen saturation and partial pressure, pH, rating of muscle soreness, and muscle damage markers, compared to the use of regular socks [10–16].

To date, the suggested beneficiary circulatory effects of wearing CS during exercise remain unclear. To our knowledge, no previous study has investigated how CS affects intramuscular pressure (IMP) and local tissue oxygenation in the anterior tibial muscle of the leg continuously, during and after running. The aim of the present study was to compare the effects of CS and regular socks on the tibialis anterior muscle, in terms of changes in IMP and muscle tissue oxygenation, and also the effects on serum biomarkers of muscle injury in healthy runners during and directly after a 10-km treadmill run.

## Methods

### Subjects

Twenty healthy runners were investigated: ten men and ten women; median age 27 (range 22‒35) years; median body weight 71 (51‒88) kg; median height 176 (161‒192) cm; and median body mass index 22 (17‒26) kg/m^2^. The inclusion criteria were as follows: able to run 10 km in 50‒60 min; good health; age 20‒60 years; and no injury to the lower legs. The subjects were involved in running activities on a regular basis, and the study was approved by the Regional Ethical Review Board. After oral and written information had been given to them, all the subjects signed an informed consent document.

### Experimental Procedures

Each subject performed two identical 10-km running sessions on a treadmill (Energetics Power Run 9.0 HRC), one session wearing CS and one session without wearing CS. The order in which the running sessions were performed was decided using a randomized crossover design. Each subject acted as his/her own control. In the running sessions without CS, the subject wore their normal ankle-high socks.

Before the first running session, the circumference of the calf was measured at the widest point of both legs for each subject, to determine the correct size guided by the manufacturer’s instructions (2XU Compression Performance Run Socks, Australia). Every subject received a new unused pair of knee-high CS. According to the manufacturer, the material of the CS was 80% nylon and 20% elastane. The compression is said by the manufacturer to be about 25 mmHg at ankle level when using the size suggested.

Each subject performed a standard warm-up, running with a speed of 8 km/h for 5 min, in the first running session and then decided a running speed between 10 and 12 km/h with no inclination for the 10-km running session. The second running session was performed with the same speed and inclination.

There were 4‒21 days between the two sessions. The variation in days is explained by the availability of the subjects, fitting the sessions in with their work or studies.

**Fig. 1:**
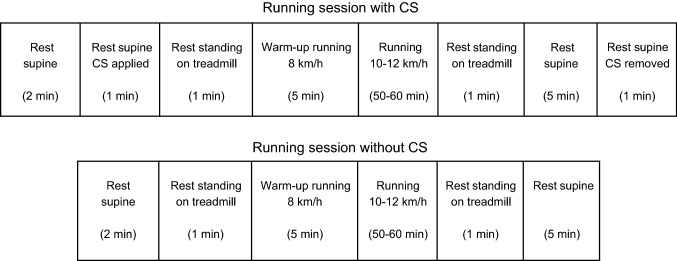
Schematic diagram of study protocol for running sessions with and without compression stockings (CS)

Measurements started with the subjects resting in supine position. This was followed by continuous measurements during the 10-km run on the treadmill and finally during a 5-min recovery period with the subjects in supine (resting) position (Fig. 1). The room temperature was between 21 and 23 °C, and the relative humidity was 23–27%.

Blood pressure was measured during the supine and standing resting positions before and after each running session. Blood samples were collected before and directly after the run (within 10 min) in the first session. In the second session, blood samples were collected only after the run.

### Measurements

#### Intramuscular Pressure

IMP was measured continuously with a catheter in the test leg of the subject using a micro-capillary infusion system (Hemo 4; Siemens, Erlangen, Germany) and monitor (Siemens SC 9000; Siemens, Gothenburg, Sweden), which have been used previously in studies on the same muscle compartment [17, 18]. The IMP measurements were randomized to the left or right leg before the test, and the same leg was monitored at both sessions for each subject. To minimize the motion artifacts of the IMP measurements, the subjects made a 5- to 15-s stop by jumping aside from the treadmill every 5 min during the running session. When the IMP recordings were made, the subjects went back on the treadmill at the same pace.

Before insertion of a pressure catheter with four side holes at its tip (IMPCATH; ProtomedLabs SAS, Marseille, France) into the anterior tibial muscle, the catheter was connected by a transducer line (200 cm) filled with saline to the pressure recording system and the monitor. Local anesthetic (2 mL of 1% lidocaine) was injected subcutaneously approximately 12 cm below the lateral condyle of the tibia and 2 cm lateral to the tibial tuberosity of the testing leg. Under sterile conditions, a Venflon introducer (1.8 × 45 mm) was inserted through the skin into the muscle in a distal direction, at an angle of 30° from the plane of the skin. The tip of the needle was retracted and the introducer bluntly advanced parallel to the fibers of the muscle, with the foot in a neutral position. Thereafter, the catheter was inserted 5 cm into the plastic sheath. The plastic sheath of the introducer was then removed.

The function of the pressure recording system was controlled by observing the response to externally applied compression of the muscle compartment and active muscle contraction by dorsiflexion of the ankle joint. The pressure recording system was calibrated according to procedures specified by the manufacturer before each measurement. The IMP value was displayed in real time on the monitor. A micro-capillary infusion technique (1.5 mL/h) was used during measurement to create a fluid pathway and establish the catheter patency. The position of the catheter was controlled by ultrasound. The median depth of the IMP catheter insertion was 14.3 (4.4‒18.8) mm in the subjects running with CS and 13.6 (4.4‒22.1) mm when they ran with normal ankle socks.

#### Muscle Tissue Oxygenation Index (TOI)

The transport of oxygen and its availability in an exercising muscle are basic factors that influence exercise performance [6]. Oxygenation of muscle tissue is directly related to the balance between oxygen consumption and oxygen supply. Near-infrared spectroscopy (NIRS) provides continuous information about the absolute level of muscle oxygenation in an exercising muscle [17, 19]. The NIRS probe unit consists of two detector photodiodes and three laser-emitting diodes held 4 cm apart. The probe was placed and affixed using double-sided adhesive tape centrally over the anterior tibial muscle of the test leg at the same position as the IMP catheter was placed. All NIRS signals were sampled at 2 Hz. Tissue oxygenation of the anterior tibial muscle was quantified using a three-wavelength, continuous-wave NIRS system (Niromonitor NIRO-200; Hamamatsu Photonics, Hamamatsu, Japan). This system simultaneously uses the modified Beer–Lambert and spatially resolved spectroscopy methods to determine concentration changes in oxygenated hemoglobin, deoxygenated hemoglobin, and total hemoglobin, all in real time. The device presents the tissue oxygen saturation in terms of the tissue oxygenation index (TOI), which expresses the ratio between oxygenated hemoglobin and total hemoglobin in the tissue under observation. The TOI value observed at the initial resting period was used as the baseline value. All subsequent data were normalized by dividing their baseline values and multiplying by 100, thus giving an initial value of 100% for normalized data.

#### Blood Pressure

Systolic and diastolic blood pressures were measured before and after the run using a pressure manometer (NAIS; Matsushita Electronic Works, Kadoma-shi, Japan), which was applied to the left forearm.

#### Blood Sampling and Analysis

It is well established that unaccustomed use of strenuous exercise induces muscle damage [20]. Both serum CK and myoglobin reflect muscle membrane disruption and have been used as biological markers to detect such damage [20, 21]. With subjects in supine position before and after the run, blood samples were collected by a nurse and sent to the routine hospital laboratory for analysis. The normal reference values for serum myoglobin are < 90 µg/L. The normal reference intervals for serum CK are 0.6‒3.5 µkat/L in women and 0.6‒6.7 µkat/L in men. Blood samples were collected from each subject before insertion of the IMP catheter and immediately after completion of the experiment (within 10 min). The modular analyzer used was Cobas 8000: module E602 for serum myoglobin and module C502 for serum CK.

#### Ultrasound Imaging

The skin and subcutaneous thickness above the anterior tibial muscle fascia and the distance between the fascia and the tip of the IMP catheter were measured using ultrasound with a linear probe (L10-5, Acuson CV70; Siemens Medical Solutions USA Inc., Malvern, PA), with the subjects in supine position and with muscles relaxed [22].

### Statistical Analysis

Paired *t* test was used to identify statistical differences in two means for IMP and TOI values between the two running sessions (with CS vs without CS). Non-parametric Wilcoxon signed rank test was used to compare matched pairs for serum myoglobin and CK concentrations. For these parameters, the significance level was set at *p* < 0.05. Statistical analysis was performed with IBM SPSS Statistics 20.

## Results

### Intramuscular Pressure

**Fig. 2:**
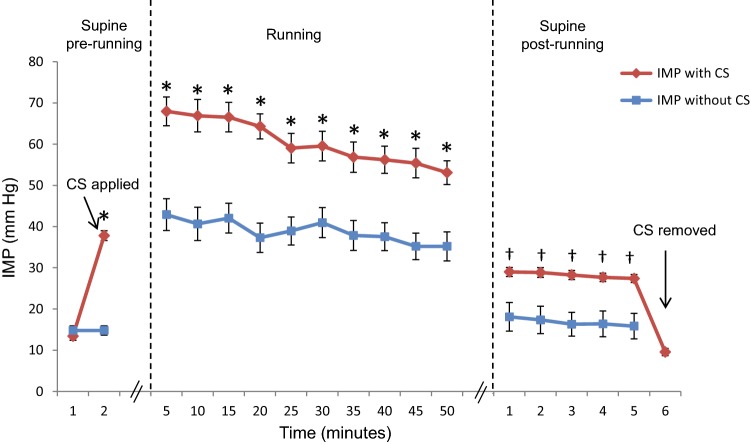
Changes in IMP in the anterior compartment before, during, and after running with or without CS. The values are expressed as mean ± standard error (*n* = 20 subjects). *CS* compression stockings, *IMP* intramuscular pressure. *p < 0.001 between the two running sessions. ^†^*p* < 0.01 between the two running sessions

Applying CS at rest elevated the baseline IMP by 24 ± 1.1 mmHg compared to the initial value (*p* < 0.001). During the running tests, the IMP values were 22 ± 3.1 mmHg higher on average when running with CS than when running without CS (*p* < 0.001). During the post-run period, the IMP values returned towards the baseline values at both running sessions, but they remained significantly higher throughout the post-run period when CS were used (*p* < 0.01). Figure 2 shows the changes in IMP over time.

**Fig. 3:**
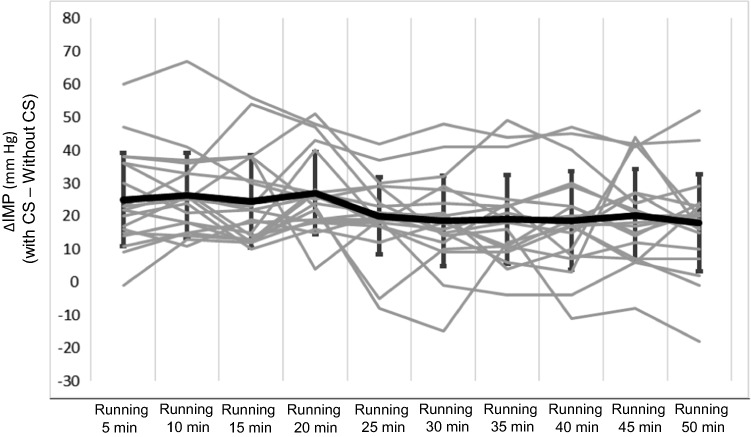
The individual differences in IMP values in the anterior compartment between running with CS and running without CS (*n* = 20). The *gray lines* denote individual subjects’ results, and the *black thick line* denotes the group mean with standard deviation values. *CS* compression stockings, *IMP* intramuscular pressure

The individual differences in IMP values between running with CS and running without CS are shown in Fig. 3. The use of CS during running caused an average increase in IMP of between 10 and 50 mmHg in the same individuals. The differences between the IMP when wearing CS or not were similar for women and men.

### Muscle TOI

**Fig. 4:**
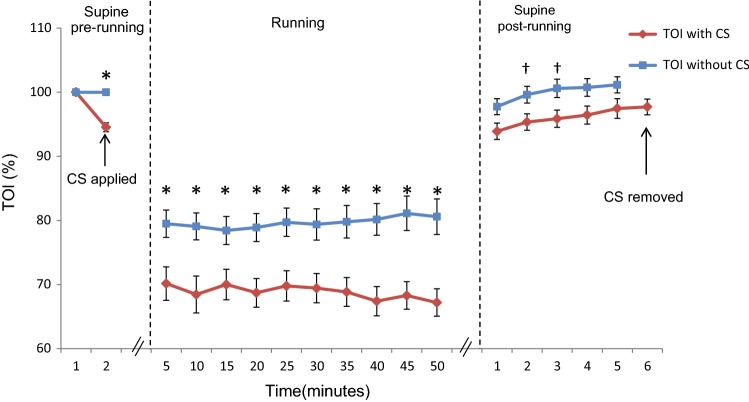
Percentage changes in TOI in the anterior compartment before, during, and after running with or without CS. The values are expressed as mean ± standard error (*n* = 20 subjects). *CS* compression socks, *TOI* tissue oxygenation index. *p < 0.001 between the two running sessions. ^†^*p* < 0.05 between the two running sessions

Applying CS at rest decreased the baseline TOI by 6 ± 0.7% (*p* < 0.001). During the running tests, the TOI values decreased in all subjects, whether running with or without CS. The mean TOI value was 11 ± 1.8% lower when the subjects were running with CS than when running without CS (*p* < 0.001). The TOI values returned towards baseline during the post-running period in both running sessions. Figure 4 shows the changes in muscle oxygenation over time relative to the baseline values. As for IMP, no gender differences were observed for changes in TOI.

### Blood Pressure

Blood pressures measured under various conditions are summarized in Table 1. No significant differences in these values were found for subjects running with CS and without CS.

**Table 1:** Outcome results for blood pressure (mmHg)

Conditions	Running with CS: systolic, diastolic	Running without CS: systolic, diastolic	*p* value^a^ BP (systolic–diastolic) with CS vs without CS
Pre-running			
Rest supine	117 ± 10.2, 74 ± 6.7	115 ± 9.2, 71 ± 7.1	0.643
Standing on treadmill	126 ± 15.0, 82 ± 10.3	126 ± 12.1, 84 ± 12.1	0.417
Post-running			
Standing on treadmill	128 ± 17.4, 65 ± 6.4	128 ± 16.3, 68 ± 7.2	0.440
Rest supine	116 ± 9.1, 65 ± 7.1	116 ± 10.3, 65 ± 6.1	0.599

Values are expressed as mean ± standard deviation

*BP* blood pressure, *CS* compression stockings

^a^For difference in BP between subjects running with and without CS

### Blood Markers for Muscle Damage

The Δ change in median serum myoglobin concentration (*n* = 20) measured before and after running was significantly greater when wearing CS [58 (9‒210) µg/L] than when not wearing CS [38 (0‒196) µg/L] (*p* = 0.04). A serum myoglobin concentration of more than 90 µg/L after running was observed in 50% of the subjects running with CS and in 25% of the subjects running without CS.

The Δ change in median serum CK concentration (*n* = 13) measured before and after running was 0.7 (− 17.6 to 6.0) µkat/L and 0.6 (− 9.3 to 6.0) µkat/L in subjects running with and without CS, respectively. No significant difference in these values was found between the two running sessions. Due to misunderstandings between the laboratory and test personnel, complete CK analyses were performed in only 13 of the 20 subjects.

## Discussion

The present study showed non-beneficial muscle effects of CS during running: increased IMP, decreased tissue oxygenation, and increased serum myoglobin. In the subjects tested, the use of CS during a 10-km treadmill run caused on average a 22-mmHg increase in IMP compared to running without CS. The increase in IMP subsequently led to a measurable reduction in muscle tissue oxygenation. Furthermore, wearing CS did not prevent exercise-induced muscle damage, as serum myoglobin significantly increased after running, after using CS. To our knowledge, this is the first study to demonstrate the effects of wearing CS on continuous measurements of IMP and muscle tissue oxygenation during and after exercise.

Before applying CS, the initial rest IMP values in both running sessions were similar and in the range of those reported by Alimi et al., who used the same method as we used to measure IMP in healthy subjects in the supine position [23]. As expected, applying CS increased the initial rest IMP by 24 mmHg on average and the IMP was significantly higher during the running test in the subjects running with CS than running without CS. Our observations support previous findings that more than 90% of externally applied pressure is transmitted directly to the muscular compartments [24]. The muscles of the anterior compartment, which were monitored in the present study, are contained in an unyielding osteofascial compartment. During application of external compression, the muscle here responded with an increase in IMP due to the compartment’s inability to expand.

This study showed that wearing CS resulted in a significant decrease in muscle oxygenation during the run, compared to running without CS. The muscle oxygenation results obtained in the present study contradict the previous findings that externally applied compression appeared to improve muscle oxygenation in the medial gastrocnemius muscle at rest [5], during exercise [6], and at rest before and after exercise [7]. The exercise protocol in the study by Coza et al. [6] was 2-min heel raises. It cannot be compared to that in the present study, where subjects performed a 10-km run at a pace of 10‒12 km/h. Bringard et al. [5] showed positive effects of CS on muscle oxygenation during rest, but this is not comparable with the present study where their effect during running was examined. Moreover, Menetrier et al. [7] found a significant increase in calf muscle oxygenation with CS at rest before and 5‒30 min after exercise. However, the response in muscle oxygenation to CS may differ between different leg muscles, due to the previously discussed anatomical differences. This may explain the adverse effects of CS on oxygenation of the anterior tibial muscle.

Blood pressures measured before and after running were similar in subjects running with CS and in the same subjects running without CS. The lower delivery of oxygen to the muscular tissue in subjects running with CS (compared to without CS) can be explained by a higher IMP in the anterior compartment. Our observations support previous findings that compartmental oxyhemoglobin saturation strongly reflects compartment pressure where muscle oxygenation decreases with increased IMP [25–28]. The present results are in line with previous findings that healthy runners do not gain any circulatory benefits from wearing CS during and after exercise [10–12, 16]. These earlier studies were done with healthy volunteers wearing CS and performing 5 min of plantar flexion exercise followed by a 5-min recovery period [11], a 40-min treadmill run [10], a half ironman triathlon race [12], or a 15.6-km trail run [16]. Castilho Junior and co-authors analyzed the effect of graduated CS on venous lower limb hemodynamics in ten healthy amateur runners [29]. They suggested wearing knee-high CS of 20–30 mmHg during a 10-km treadmill run to improve venous hemodynamics that may enhance recovery of muscle function and reduce muscle soreness. No positive effects on calf muscle pump, capillary lactate variation, and after-run heart rates were observed [29]. The present study did not investigate the venous hemodynamics.

By having the CS covering the anterior compartment, where the measurements were performed, an increase in skin temperature can be expected, compared to when using ankle-high socks. The effect of increased skin temperature on muscle oxygen levels has been investigated by Hom et al. [30]. They found a lower decrease of Oxygen saturation (StO_2_) during exercise with an increased skin temperature. Based on this it could be speculated that the effect of the CS possibly could have been underestimated because of the skin temperature rise with the CS. However, we did not investigate the skin temperature or its effect on the measurements in the present study.

One of the cornerstones in marketing CS is the claim that there is reduction of muscular damage. Our results demonstrate that wearing CS during running does not prevent exercise-induced muscle damage. Serum myoglobin measured directly after running significantly increased in subjects running with CS, and no significant difference was found in post-run values of serum CK concentrations between running with CS and running without CS. Serum myoglobin reflects the damage to both type-1 and type-2 muscle fibers [31]. Serum CK is known to be present at elevated levels after exercise, for up to 48 h, with peak values 24 h after exercise; peak values of serum myoglobin have been recorded 1 h after exercise [32]. Our post-run results of serum myoglobin and CK are limited since the blood sample was collected on only one occasion after the run. It would be of interest to explore repetitive blood samples during a longer period post-running in future studies.

The results of the present study suggest that the use of CS is ineffective in reducing muscle damage during a 10-km treadmill run. Our findings support previous reports that the use of CS does not reduce blood markers of muscle damage when running races [12, 33].

IMP increases with depth from a subfascial position to the centrally located tendon [34], and the placement of the IMP catheter may affect the values measured. This consideration did not influence the comparison of IMP values between running with or without CS in the present study, as the depth of the IMP catheter was similar, and the positioning was confirmed by ultrasound.

Although great care was taken to ensure that IMP catheters and NIRS sensors were placed in the same location in subjects running with or without CS, it is possible that the catheters and sensor positions had an influence on the results obtained. We would expect these errors to be negligible, since we used measurements based on anatomical landmarks for placement of the catheters and sensors in each experiment.

The number of subjects in the present study was 20. This number was based on the power analysis for the difference in IMP, and we did not make similar considerations for the biomarkers. A limitation of the study is that the effects of CS were investigated only in the anterior compartment and CS may have different effects on other compartments of the legs. The unblinded design and the possible placebo effect of CS can also be considered as limitations. However, since only objective measurements were obtained in the present study, no effects on the results are to be expected from these factors.

## Conclusions

Wearing exercise CS during and following a 10-km treadmill run elevated IMP and reduced muscle tissue oxygenation in the anterior compartment of healthy subjects. Furthermore, the use of exercise CS did not prevent early exercise-induced muscle damage, as measured by serum biomarkers.
